# A 60% crisis: Mortality and hypertension-driven intracranial haemorrhage at a South African tertiary hospital

**DOI:** 10.4102/jcmsa.v3i1.202

**Published:** 2025-07-28

**Authors:** Umar Jacobs, Thaakir-Ahmed Jacobs, Janneke Pienaar, Sideeqa Jakoet, Aaqilah Fataar, Neshaad Schrueder, Sa’ad Lahri

**Affiliations:** 1 Division of General Internal Medicine, Department of Medicine, Stellenbosch University, Cape Town, South Africa; 2 Division of General Internal Medicine, Department of Medicine, University of Cape Town, Cape Town, South Africa; 3 Division of Chemical Pathology, Faculty of Health Sciences, Stellenbosch University, Cape Town, South Africa; 4 Division of Emergency Medicine, Department of Family and Emergency Medicine, Stellenbosch University, Cape Town, South Africa

**Keywords:** age, gender, risk factors and comorbidities, blood pressure on admission, CT scan results, use of antiplatelet and anticoagulation medication, clinical outcome of patient

## Abstract

**Background:**

Spontaneous intracranial haemorrhage (ICH) is a significant cause of morbidity and mortality worldwide, with a disproportionate burden in low- and middle-income countries. Data on ICH in South Africa are limited, hindering targeted intervention efforts.

**Methods:**

A retrospective, descriptive study was conducted at Tygerberg Hospital, Cape Town, reviewing records of patients with confirmed spontaneous ICH based on computed tomography imaging from 01 January 2021 to 31 December 2022. Demographics, risk factors, clinical presentation, imaging findings and outcomes were analysed.

**Results:**

Of the 162 eligible cases, 53.09% were male, with a mean age of 51.81 years (standard deviation: 11.88). Hypertension was the most prevalent risk factor (87.04%), with 84.57% presenting with grade 2 hypertension on admission. Basal ganglia involvement was the most common ICH location (55.56%). Complications were frequent, with 79.82% showing intraventricular extension. The 3-month and 1-year mortality rates were 59.88% and 60.49%, respectively. Only 57.41% of hypertensive patients were on antihypertensive medication prior to ICH.

**Conclusion:**

This study highlights the significant burden of spontaneous ICH in a South African tertiary hospital setting, characterised by a younger age of onset and high prevalence of modifiable risk factors, particularly uncontrolled hypertension. These findings underscore the urgent need for enhanced hypertension management and targeted primary prevention strategies to reduce the ICH burden, providing valuable data to inform public health interventions in resource-limited settings.

**Contribution:**

This study provides data on spontaneous intracranial hemorrhage (ICH) in a South African tertiary hospital, identifying a high mortality rate and the prevalence of uncontrolled hypertension in a younger population. The findings address a key data scarcity in low- and middle-income countries and promote the development of tailored prevention and management initiatives, which are consistent with the journal’s emphasis on regionally appropriate, evidence-based healthcare interventions.

## Introduction

Spontaneous intracranial haemorrhage (ICH), defined as non-traumatic bleeding of the brain parenchyma, represents a significant global health challenge, accounting for approximately 10% of all stroke cases worldwide.^1,2^ In recent decades, the incidence of ICH has more than doubled, with mortality rates ranging from 30% to 40%.^3^ Despite the considerable functional loss and high mortality rate after ICH, care systems for people with ICH have historically trailed behind those with acute ischaemic stroke.^4^ This devastating neurological emergency not only claims lives but also leaves many survivors with severe disabilities, cognitive decline and at high risk for recurrent strokes.^3^

The burden of ICH is disproportionately distributed across the globe.^5^ The Global Burden of Disease study revealed a stark disparity between high-income countries and low- to middle-income countries (LMICs).^5^ Low- to middle-income countries, including South Africa, face a higher incidence of ICH and worse outcomes.^6^ Between 1990 and 2010, there was a significant increase in absolute stroke cases in developing countries, accompanied by a loss of approximately 80% of disability-adjusted life years (DALYs).^5,7^

In South Africa, the situation is particularly concerning because of the high prevalence of hypertension, a primary risk factor for ICH.^8^ Hypertension is caused by a complex combination of environmental, behavioural and hormonal factors, as well as many organ systems.^9^ Dysregulation of these processes often results in hypertension, which may further lead to hypertension-mediated organ damage and serious cardiovascular disease (CVD) outcomes if left untreated.^9^ World Health Organization data indicate that approximately 26.1% of women and 27.4% of men in the country have hypertension.^8,10^ More alarmingly, a meta-analysis by the Non-Communicable Diseases Risk Factor Collaboration showed that the prevalence of hypertension has doubled from 1990 to 2019, with particularly poor control in low socioeconomic settings.^5,6^

Despite the magnitude of this health threat, a critical knowledge gap persists regarding the specific burden of ICH in South Africa.^11^ While global data on ICH are well documented, comprehensive local epidemiological information is scarce.^12^ This lack of data hinders the development of targeted interventions, appropriate resource allocation and effective public health strategies. Unlike many countries that have implemented national stroke registries, South Africa lacks this vital tool for understanding and combating ICH.

Furthermore, the unique socioeconomic factors and healthcare challenges in South Africa may influence ICH presentation, management and outcomes in ways that differ significantly from high-income settings. Understanding these nuances is crucial for developing contextually appropriate interventions and policies.

This study aims to address this critical knowledge gap by providing a comprehensive analysis of spontaneous ICH in a tertiary academic hospital in South Africa. The objectives of this study intend to characterise the demographic profile of individuals with confirmed spontaneous ICH and ascertain the all-cause mortality rates among these patients at 3 months, 1 year and 3 years after the event. The study also seeks to determine the prevalence of identified risk factors for ICH, including hypertension, diabetes, dyslipidaemia, smoking, alcohol consumption, prior stroke, cardiovascular disease and human immunodeficiency virus (HIV) infection. Furthermore, it aims to determine the proportion of patients who were taking antiplatelet, anticoagulant, antihypertensive or statin drugs prior to the onset of ICH. Finally, the study aims to describe the clinical presentation, underlying aetiology and prognosis of patients with spontaneous ICH.

By examining these aspects, we seek to shed light on the true burden of this condition in our population. Our findings have the potential to not only impact clinical practice within South Africa but also contribute to the broader understanding of ICH in LMICs, where resources are limited and the need for cost-effective interventions is paramount.

As South Africa grapples with the dual burden of communicable and non-communicable diseases, insights from this study could prove invaluable in shaping public health policies and healthcare delivery strategies. By illuminating the current landscape of ICH in our context, we aim to pave the way for targeted prevention strategies, improve clinical management and, ultimately, a reduction in the devastating impact of this condition on South African lives.

## Research methods and design

This retrospective, descriptive study was conducted at Tygerberg Hospital, a 1384-bed tertiary academic hospital in Parow, Cape Town, South Africa. Tygerberg Hospital, affiliated with the University of Stellenbosch, serves a population of over 3.4 million people across six drainage areas.^13^ The study period spanned from 01 January 2021 to 31 December 2022.

The study population included all adult patients presenting to Tygerberg Hospital with confirmed spontaneous ICH based on computed tomography (CT) imaging during the study period. Exclusion criteria encompassed traumatic ICH; secondary causes of ICH (including subarachnoid, subdural and extradural haemorrhages, arteriovenous malformation [AVM] and aneurysms); incomplete medical records; unconfirmed stroke; haemorrhagic cerebral tumours; acute ischaemic stroke (with or without haemorrhagic transformation) and cerebral venous thrombosis.

The sample was calculated at 161 participants using WINPEPI software, based on a 1-year survival rate of 46% after spontaneous ICH in upper-middle-income countries.^14^ This sample size allows for an estimation of the survival rate with a precision of ±8% at a 95% confidence interval.

Patient records were identified using the ICD-10 coding system from the hospital’s information management unit. Computed tomography imaging reports were accessed from the Picture Archiving and Communication System (PACS) database, and medical records were reviewed using the Enterprise Content Manager (ECM) system. Data collected included demographics, comorbidities, risk factors, medication history, ICH characteristics and outcomes.

Data management and quality control data were recorded on standardised collection sheets (see Appendix 1) and entered into a secure online Google Forms database. Each patient was assigned a unique study number to ensure anonymity. To maintain data integrity, 10% of entries were randomly selected for cross-checking by a second researcher. Furthermore, mortality statistics were obtained through patient records on ECM and subsequently confirmed via the single patient viewer online system.

### Statistical analysis

Data analysis was performed using Microsoft^®^ Excel for MAC, version 17 (StataCorp, Texas, United States) and R4.3.1 with RStudio and survival and ggplot2 packages. Incomplete data points were excluded from the analysis. Descriptive statistics were used to summarise all variables. Categorical data were presented as frequency counts and percentages. Continuous variables were described using means with standard deviations (s.d.) or medians with interquartile ranges, as appropriate.

For survival analysis, Kaplan–Meier curves were constructed to display cumulative survival rates at 3 months and 1-year post-ICH. Cox proportional hazards regression was used to identify factors associated with mortality, adjusting for potential confounders.

All statistical tests were two-sided, with a *p*-value < 0.05 considered statistically significant. Where applicable, 95% confidence intervals were calculated to indicate the precision of estimates.

### Ethical considerations

Ethical clearance to conduct this study was obtained from the Stellenbosch University Health Research Ethics Committee (No. S23/03/045). Tygerberg Hospital granted permission for the use and analysis of patient records. Given the retrospective nature of the study, the requirement for individual patient consent was waived. All data were managed in compliance with ethical guidelines to ensure patient confidentiality. Patient information and records will be stored safely on the primary researcher’s computer that is password and username protected. All data collected will be allocated a specific study number and saved in a password-protected folder. Patients’ folder numbers will then be placed in a separate folder that is also password protected. Information pertaining to this study will only be allowed access to me and respective supervisors.

## Results

A total of 615 patient records were reviewed based on specific type and location of ICH; however, only 162 cases were eligible for the study and included in the analysis. The remainder of the records was excluded based on either missing data, alternative diagnoses to spontaneous ICH, or incomplete CT scan reports.

Of the 162 cases included in the study, 86 (53.09%) were male and 76 (46.91%) were female. The ages ranged from between 26 years and 82 years, with an average age of 52 years (mean (s.d.) 51.81 (11.88) years).

**FIGURE 1:**
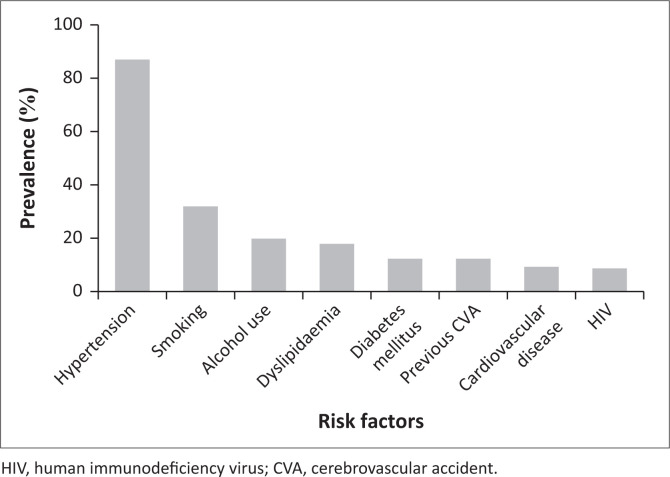
Risk factors associated with spontaneous intracranial haemorrhage.

Regarding patient comorbidities and risk factors for spontaneous ICH, hypertension (87.04%) was the leading and most common risk factor identified in the sample. Furthermore, as noted from the sample, class 2 hypertension (84.57%) was found as the most predominant blood pressure (BP) on admission, indicating the severity of BP in patients with haemorrhagic strokes. Patients who were smokers (32.1%) were noted to be the second most common risk factor, following alcohol abuse (19.75%) and dyslipidaemia (17.90%) (See Table 1 and Figure 1).

**TABLE 1:** Comorbidities and risk factors for the study population.

Comorbidities and risk factors	*n*	%
Hypertension	141	87.04
Diabetes mellitus	20	12.35
Dyslipidaemia	29	17.90
Smoking	52	32.10
Alcohol use	32	19.75
Previous CVA	20	12.35
Cardiovascular disease	15	9.26
HIV	14	8.64

HIV, human immunodeficiency virus; CVA, cerebrovascular accident.

From the studied population, most patients had between 1 and 3 risk factors (82.10%) for ICH. Notably, BP on admission for the study population showed a significantly higher proportion of patients with grade 2 hypertension BP > 160/100 mmHg (*n* = 137, 84.57%), with a total of 9 (5.56%) having grade 1 hypertension, 4 (2.47%) having high normal BP of 130–139/85–89 mmHg and 12 (7.41%) having normal BP on admission.

All CT scans that were reported by radiologists had documented ICH of various locations. Ninety (55.56%) had basal ganglia involvement, with the thalamus (23 (14.20%)) being the second most common location of haemorrhagic stroke seen.

There were also a total of 15 (9.26%) patients who had > 1 region of the brain being affected. Moreover, of the sample population studied, 114 (70.37%) had notable evidence of complications from their ICH; for example, intraventricular extension in 91 (79.82%) and herniation syndrome in 77 (67.54%) (see Table 2).

**TABLE 2:** Location of the intracranial haemorrhage.

Region of brain affected	*n*	%
Frontal lobe	18	11.11
Parietal lobe	19	11.73
Occipital lobe	4	2.47
Temporal lobe	13	8.02
Basal ganglia	90	55.56
Thalamus	23	14.20
Deep cerebellum	6	3.70
Brainstem	14	8.64
**Complications**		
Intraventricular extension	91	79.82
Herniation syndrome	77	67.54

**FIGURE 2:**
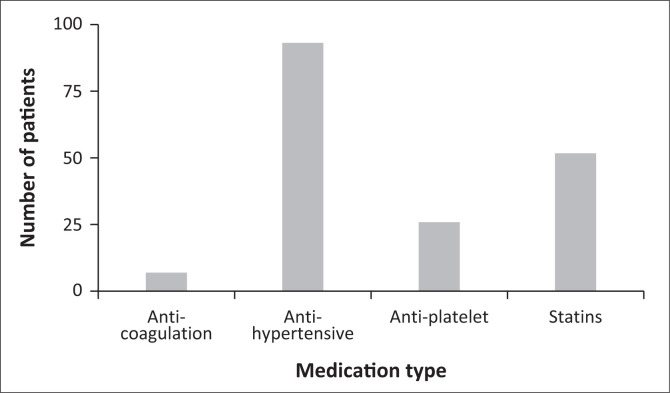
Distribution of medication usage in patients with ICH.

Medication used prior to the development of an acute haemorrhagic event was analysed to assess for non-adherence and how this may have impacted their event. The study shows that patients who had hypertension (87.04%), a total of 93 (57.41%) were on antihypertensive medication. Likewise, 7 (4.32%) were using either warfarin/rivaroxaban, 26 (16.05%) were on antiplatelet therapy with either aspirin or clopidogrel and 52 (32.10%) had been on a statin (see Figure 2).

**FIGURE 3:**
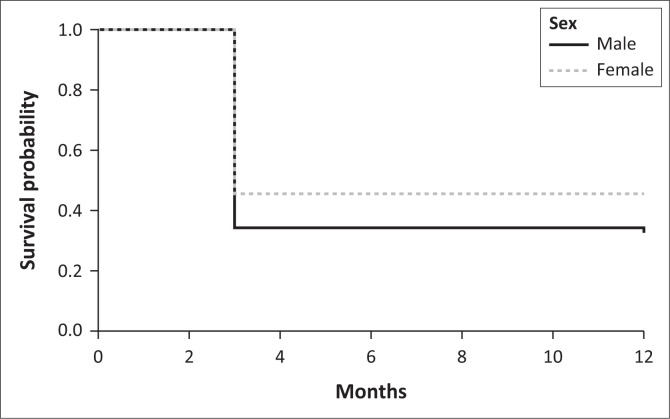
Kaplan–Meier survival curve.

Mortality following spontaneous ICH was assessed (Figure 3), revealing that 97 patients (59.88%) died within 3 months of the event. An additional patient succumbed to their condition 12 months post-ICH. Of the deceased patients, 51 were female, with the patient who died at 12 months also being female.

## Discussion

This retrospective study provides valuable insights into the epidemiology, clinical characteristics and outcomes of spontaneous ICH in a South African tertiary care setting. Our findings highlight the significant burden of ICH in this population and underscore the urgent need for improved prevention and management strategies.

Our study revealed a slight male predominance (53.09%) in ICH patients, which is consistent with previous studies in both developed and developing countries. The incidence of ICH in men is higher than in women, as indicated by this study, partly because of the difference being closely related to age; however, dietary practices, genetics, social pressure and a host of other factors all play a major role.^15^ The mean age of 51.81 years (s.d. = 11.88) is notably lower than that reported in high-income countries, where the average age of ICH onset is typically in the sixth or seventh decade of life.^16^ This younger age of onset in our population is particularly concerning, as it affects individuals in their prime working years, potentially leading to significant socioeconomic consequences.

The wide age range (26–82 years) observed in our study population underscores the need for heightened awareness of ICH risk across all adult age groups. It also highlights the importance of early screening and management of risk factors, even in younger adults, particularly in LMICs like South Africa.

Hypertension emerged as the dominant risk factor, present in 87.04% of our patients. This prevalence is higher than that reported in many international studies,^6,16^ reflecting the significant burden of uncontrolled hypertension in South Africa. The fact that 84.57% of patients presented with grade 2 hypertension (BP > 160/100 mmHg) on admission is alarming and suggests either poor BP control in the community or acute hypertensive responses to the ICH event.

The high prevalence of other modifiable risk factors such as smoking (32.1%), alcohol abuse (19.75%) and dyslipidaemia (17.90%) indicates potential targets for public health interventions. The relatively low prevalence of diabetes mellitus (12.35%) in our cohort is interesting and warrants further investigation, as it differs from patterns seen in some other populations.

The predominance of basal ganglia haemorrhages (55.56%) in our study is consistent with hypertensive vasculopathy being the primary underlying pathology. This pattern is typical in populations with a high prevalence of hypertension and differs from that seen in populations where cerebral amyloid angiopathy is more common, which tends to result in more lobar haemorrhages.^16,17,18^

The high rates of complications, particularly intraventricular extension (79.82%) and herniation syndrome (67.54%), are concerning and likely contribute to the poor outcomes observed. These findings emphasise the need for rapid diagnosis, transfer to appropriate care facilities and aggressive management of ICH in our setting.

Our analysis of pre-ICH medication use revealed important gaps in preventive care. Despite the high prevalence of hypertension, only 57.41% of patients were on antihypertensive medication. This discrepancy highlights significant challenges in hypertension awareness, treatment and control in our population. The relatively low use of anticoagulants (4.32%) and antiplatelet agents (16.05%) may reflect differences in the prevalence of atrial fibrillation and ischaemic heart disease compared to high-income countries or potentially underdiagnosis of these conditions.

The high short-term mortality rates observed in our study (59.88% at 3 months and 60.49% at 1 year) are higher than those reported in many high-income countries but are consistent with data from other LMICs.^19,20^ These findings highlight the high early mortality rate associated with spontaneous ICH, with a notable proportion of female patients among those who did not survive.

The Kaplan–Meier survival curve reveals a rapid decline in survival probability within the first 3 months following spontaneous ICH, which corresponds to the high mortality rate observed in this period. This early drop in the curve reflects the acute mortality associated with ICH, where most patients who succumb do so within the first few months following the event. These findings underscore the high risk in the early post-ICH period and highlight the need for more granular follow-up data for a comprehensive understanding of long-term survival patterns.

These poor outcomes likely reflect a combination of factors, including delayed presentation, severity of ICH at presentation and potentially limited resources for acute management and rehabilitation.

Our findings underscore the critical need for improved primary prevention strategies in South Africa, particularly focusing on hypertension control. Community-based interventions for BP screening, lifestyle modification programmes and initiatives to improve medication adherence could significantly impact ICH incidence and outcomes.

The younger age of ICH onset in our population compared to high-income countries suggests that prevention efforts should start earlier in life. Public health campaigns to increase awareness of stroke symptoms and risk factors should target a broad age range, including young adults.

The high complication rates and poor outcomes observed in our study highlight the need for systemic improvements in acute stroke care. This may include optimising prehospital care, establishing more dedicated stroke units and improving access to neurosurgical interventions when appropriate.

### Limitations

This study has several limitations that should be considered when interpreting its results. As a retrospective review, it inherently carries the limitations of this study design. Missing and incomplete data were inevitable, which may have introduced bias into our findings. To mitigate this, we meticulously documented all excluded cases because of limited or incomplete records in a separate file, allowing for comparison with the study population. This approach helps to assess the potential impact of excluded data on our results.

While we collected data over a 2-year period to increase the sample size and enhance the robustness of our findings, the study was conducted at a single tertiary centre. This may limit the generalisability of our results to other settings in South Africa, particularly primary and secondary healthcare facilities or rural areas. Because of limitations in data collection regarding mortality statistics, where the outcome was assessed only at the 3-month and 1-year marks, exact event dates within this period were not recorded, contributing to the abrupt nature of the drop seen in the curve. Additionally, data were censored at 1 year, as no follow-up information was available beyond this point, resulting in an artificial plateau in the curve. This approach, while providing a snapshot of survival rates, limits detailed analysis of survival timing and further limits our ability to assess long-term functional outcomes and quality of life among survivors of ICH.

Our reliance on medical records and imaging reports means that we were limited to the information documented by healthcare providers at the time of patient care. This unfortunately included limitations to the types of treatment and intervention patients had received at the time of presentation. This could have led to underreporting of certain risk factors or complications that were not explicitly recorded.

While we identified various risk factors associated with ICH, our study design does not allow for the establishment of causal relationships. Further prospective studies would be needed to confirm the causality of these associations. Our study did not include a control group, which limits our ability to compare the prevalence of risk factors in ICH patients with the general population in the same catchment area.

Despite these limitations, our study provides valuable insights into the epidemiology and clinical characteristics of spontaneous ICH in a South African tertiary care setting, contributing to the limited body of research on this topic in sub-Saharan Africa.

## Conclusion

This study underscores the substantial burden of spontaneous ICH in a South African tertiary setting, revealing critical areas for intervention. The high prevalence of severe hypertension and the relatively young age of onset highlight an urgent need for enhanced hypertension management and screening programmes. Predominant basal ganglia involvement, frequent complications and high mortality rates further emphasise the severity of ICH presentations in this population. Our findings call for a multi-faceted approach to address this health crisis. Implementing targeted community-based interventions for hypertension control, increasing public awareness about stroke risk factors and strengthening acute stroke care services are essential steps. The gaps identified in preventive medication use, particularly antihypertensives, suggest a need for strategies to improve treatment adherence and follow-up care.

Future research should focus on prospective, multi-centre studies to further elucidate risk factors, treatment outcomes and long-term prognosis of ICH across diverse South African healthcare settings. Evaluating the effectiveness of targeted interventions to reduce ICH incidence and improve outcomes is crucial. By addressing these challenges, there is significant potential to reduce the impact of ICH on individuals, families and the healthcare system in South Africa, ultimately improving public health outcomes in the region.
